# Stereological Study on the Positive Effect of Running Exercise on the Capillaries in the Hippocampus in a Depression Model

**DOI:** 10.3389/fnana.2017.00093

**Published:** 2017-11-17

**Authors:** Linmu Chen, Chunni Zhou, Chuanxue Tan, Feifei Wang, Yuan Gao, Chunxia Huang, Yi Zhang, Lin Jiang, Yong Tang

**Affiliations:** ^1^Department of Histology and Embryology, Chongqing Medical University, Chongqing, China; ^2^Department of Geriatrics, The First Affiliated Hospital, Chongqing Medical University, Chongqing, China; ^3^Department of Physiology, Chongqing Medical University, Chongqing, China

**Keywords:** capillaries, depression-like behavior, hippocampus, exercise, stereology

## Abstract

Running exercise is an effective method to improve depressive symptoms when combined with drugs. However, the underlying mechanisms are not fully clear. Cerebral blood flow perfusion in depressed patients is significantly lower in the hippocampus. Physical activity can achieve cerebrovascular benefits. The purpose of this study was to evaluate the impacts of running exercise on capillaries in the hippocampal CA1 and dentate gyrus (DG) regions. The chronic unpredictable stress (CUS) depression model was used in this study. CUS rats were given 4 weeks of running exercise from the fifth week to the eighth week (20 min every day from Monday to Friday each week). The sucrose consumption test was used to measure anhedonia. Furthermore, stereological methods were used to investigate the capillary changes among the control group, CUS/Standard group and CUS/Running group. Sucrose consumption significantly increased in the CUS/Running group. Running exercise has positive effects on the capillaries parameters in the hippocampal CA1 and DG regions, such as the total volume, total length and total surface area. These results demonstrated that capillaries are protected by running exercise in the hippocampal CA1 and DG might be one of the structural bases for the exercise-induced treatment of depression-like behavior. These results suggest that drugs and behavior influence capillaries and may be considered as a new means for depression treatment in the future.

## Introduction

Depressive disorder is widely distributed in the population (Kessler et al., 2003), as it is the main reason of disability worldwide (Menard et al., 2016). Thus far, the depression theories of inflammatory (Maes et al., 2012), monoamine (Blier, 2003) and neurotrophic (Duman and Monteggia, 2006) compounds have been introduced by major researchers. Common antidepressant drugs act on monoamine neurotransmitters because of their significant role in the pathogenesis of depression. However, Malki et al. (2012) found that lag time always exists between the improvement of depressive symptoms and the restoration of the neurochemical balance, which means that the drug response of the primary targets might have comprehensive downstream biological events and that further studies are needed. To provide a new treatment for depression, it is important to further study the pathogenesis of depression.

Positive benefits in cognitive, emotional and motor domains combined with reductions in negative affect and distress can be acquired with regular physical exercise (Archer et al., 2014). Greist et al. (1979) revealed that aerobic running is a treatment for moderate depression. Some studies revealed that physical exercise might be a safe and effective antidepressant therapy in late-life major depression patients (Belvederi Murri et al., 2015) and in rats and mice with depression-like behavior (Lee et al., 2013; Eldomiaty et al., 2017). However, the mechanisms underlying the antidepressant effect of running exercise are not fully understood.

Blood vessels can be influenced by running exercise (Bolduc et al., 2013). Cerebrovascular benefits can be gained with regular engagement in physical activity (Guiney et al., 2015). The positive effects of running exercise on cerebral capillaries have been investigated by many studies. Kleim et al. (2002) found that independent running training for 30 days increased the capillary density of motor cortex in the 5-month-old male rats. Swain et al. (2003) found that after 30 days of running exercise, in 6- to 12-month-old rats the volume fraction of the cortex capillaries is increased in the II/III layer of the motor cortex. Using unbiased stereological methods, our previous work revealed that after running exercise the total length, total volume and total surface area of the capillaries in the cortex of aged rats (Huang et al., 2013) and in white matter in a rat model of depression (Chen et al., 2016) are increased. In treating vascular depression, greater depression reduction and lower rates of recurrence are found when combined antidepressant therapy with vasodilator drug (nimodipine), which supported the argument that cerebrovascular disease is related to the pathogenesis and recurrence of depression in patients (Taragano et al., 2001). As mentioned above, the change of capillaries might be involved in the pathogenesis of depression, and running exercise may reverse the symptoms of depression.

The knowledge gained from imaging research and the postmortem studies is catalyzing a paradigm shift in which primary mood disorders are conceptualized as illnesses that involve abnormalities in brain structure and function (Drevets, 2001). The hippocampus has a significant role in the feedback inhibition of the stress response (Yang et al., 2003). Autopsy results of depression patients confirm changes in the hippocampus (Duman et al., 2000). Using MRI, researchers found no significant difference in the whole brain volume between depression patients and normal individuals, but the hippocampal volume was significantly smaller in depression patients than in normal individuals (Nifosi et al., 2010; Ahdidan et al., 2011; Amico et al., 2011). Therefore, the hippocampus plays an important role in the process of depression, but the specific mechanism remains to be further studied.

Nevertheless, whether vascular change in hippocampus is involved in the treatment effect of running exercise on depression-like behavior in rat is still unknown. We hypothesized that the microvascular system in the hippocampus in depression model undergoes significantly change before and after running exercise. To test this hypothesis, we established an effective and feasible depression animal model via chronic unpredictable stress (CUS) stimuli (Manosso et al., 2016). After running exercise, a stereological quantitative method was used to investigate the changes in capillaries in hippocampal CA1 and dentate gyrus (DG) regions in a rat model of depression.

## Materials and Methods

### Animals

The Experimental Animal Center of Chongqing Medical University provided the male Sprague-Dawley rats. The rats were acclimated to their surroundings for 1 week before experimentation. They were individually housed at a temperature of 22 ± 2°C, a humidity of 55 ± 10% and kept under a constant 12 h light-12 h dark cycle. According to the open field test, 100 rats were picked up for the next experiment. This study was carried out in accordance with the recommendations of Chongqing Management Approach of Laboratory Animal (Chongqing government order No.195). The protocol was approved by the Institutional Review Board of Chongqing Medical University (Reference Number: CQMU 2010–26).

### Establishment of the Depression Model and Running Exercise Protocol

The main CUS process was described in a previous study (Chen et al., 2016). The basic steps are as follows: the rats were randomly divided into two groups: control group (*n* = 10) and CUS group (*n* = 90). Each rat in the CUS group was given CUS stimuli while housed in a single cage. The rats in the control group were housed 5 rats per cage and were not given CUS stimuli. Each random stimulus did not appear continuously. Then, 20 rats with significantly lower sucrose consumption were selected from the CUS group and divided into a CUS/Standard group (*n* = 10) and a CUS/Running group (*n* = 10). The rats in the CUS/Running group were forced to run on a six-lane motorized treadmill. The exercise protocol was similar to that of a previous study (Chen et al., 2016).

### Behavior Test

Anhedonia, the core symptom of depression in humans (Scheggi et al., 2015), can be measured via a sucrose consumption test (Hurley et al., 2014). The detailed protocol was described in a previous report (Hurley et al., 2014). The sucrose consumption test was carried out every Saturday at 7 am for 8 weeks.

The general exploratory behavior and locomotor behavior were evaluated by open field test. The test was operated as described previously with modifications (Duman et al., 2008).

### Tissue Processing

First, the rats were anesthetized with 1% pentobarbital sodium (4 ml/kg). Then, 4% paraformaldehyde was diluted with PBS (pH = 7.4), and 0.9% heparinized saline was perfused intracardially in left ventricular apex in sequence. After that, the cerebrum was obtained and cut into two hemispheres. Each hemisphere was sliced into 1 mm consecutive slabs coronally.

### Estimation of the CA1 and DG Region Volumes

**Figure 1:**
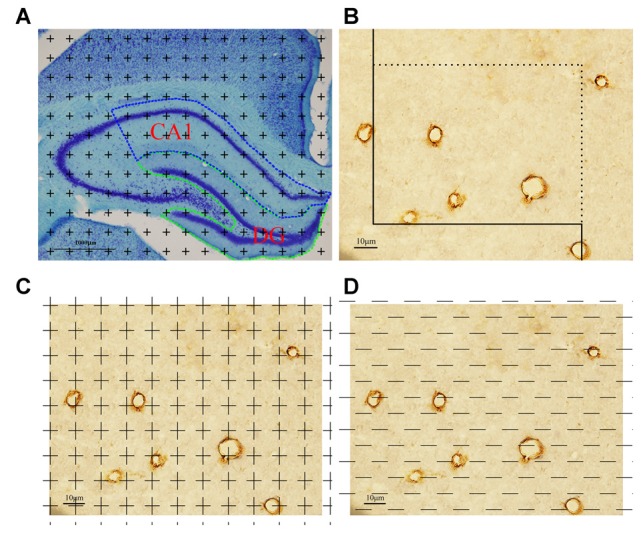
Graphical annotation of stereology methods. **(A)** Toluidine blue staining was used to reveal the hippocampus partition. CA1 and dentate gyrus (DG) regions are surrounded by the blue and green lines, respectively (4×, Scale bar = 1000 μm). **(B)** The calculation method of capillary length was as follows: each captured image was put on an unbiased counting frame. The capillary profiles would be counted if they are completely inside the counting frame or touched the counting lines (solid lines); otherwise, they would not be counted. Scale bar = 10 μm. **(C)** The calculation method of capillary volume was as follows: each captured image was put on unbiased point grid. The capillaries would be counted when they hit the points. Scale bar = 10 μm. **(D)** The calculation method of capillary surface area was as follows: each captured image was put on unbiased test lines. The capillaries would be counted when they had intersections with the test lines. Scale bar = 10 μm.

One 10-μm-thick frozen slice was obtained from each 1 mm slab. Toluidine blue staining was done to mark the area of CA1 and DG region (Figure 1A). The total CA1 and DG region volumes in each 1 mm slab were calculated through Cavalieri’s principle (Chao et al., 2015). A brief description is as follows: the caudal surface of each slab was hit by randomly equidistant points in the transparent plastic sheet and the points hitting the object region were counted. The calculation formula was as follows (Yang et al., 2015):

(1) $${\text{V}}_{\left(\text{CA1}\right)}=\text{t}\times \text{a}(\text{p})\times \sum {\text{P}}_{\left(\text{CA1}\right)}\times 2$$

t means the slab thickness (1 mm), a(p) means the area associated with each point and ∑P_(CA1)_ means the total number of points hitting the CA1 per hemisphere.

The DG region volume was similarly calculated using the above methods.

### Sampling from the CA1 and DG Regions and Immunohistochemical Staining

Sampling methods were described by our previous work (Chen et al., 2016). The obtained slabs were embedded in optimal cutting temperature compound (OCT) to obtain frozen sections, and the sector technique was used to produce isotropic and uniformly random (IUR) sections (Chao et al., 2015). The thickness of the frozen section is 4 μm. On average, 20 frozen sections could be obtained per rat.

A brief description of immunohistochemical staining is as follows: each slice was fixed in acetone for 10 min and put in a high temperature in citrate solution for 15 min to repair antigens. Then, 3% hydrogen peroxide for 15 min at 37°C was used to inactivate endogenous peroxides, and nonspecific staining was blocked by normal goat serum for 30 min at 37°C. Mouse monoclonal CD31+ (Abcam, UK; Albelda et al., 1991) was incubated at 4°C overnight. Subsequently, the slice was transferred to a specific secondary antibody solution for 30 min at 37°C and diaminobenzidine (DAB) solution was used to display color. After the above procedure, the section was observed using a light microscope of a stereological system. The vessels with a luminal diameter of less than 10 μm were defined as components of the capillary network (Alba et al., 2004).

### Total Length of the Capillaries in CA1

Every captured photograph was randomly superimposed by an unbiased counting frame (Figure 1B). The length density of the capillaries in the CA1 was analyzed with a method previously described (Shao et al., 2010):

(2) $${\text{L}}_{\text{V}}(\text{cap}{/}_{\text{CA1}})=2\times \sum \text{Q}(\text{cap}{/}_{\text{CA1}})/\sum \text{A}$$

ΣQ (cap/_CA1_) means the total number of the capillary profiles in CA1 counted per rat. ΣA means the total area of the counting frames used per rat. The total length of the capillaries in the CA1 could be obtained by multiplying the length density of the capillaries in the CA1, L_V_ (cap/_CA1_), by the CA1 volume (V_(CA1)_).

### Total Volume of the Capillaries in CA1

Every captured photograph was randomly placed by a transparent point grid (Figure 1C). The volume fraction of the capillaries in the CA1, V_V_ (cap/_CA1_), was analyzed using the Following formula (Shao et al., 2010):

(3) $${\text{V}}_{\text{V}}(\text{cap}{/}_{\text{CA1}})=\sum \text{P}(\text{cap}{/}_{\text{CA1}})/\sum \text{P}{(}_{\text{CA1}})$$

The total volume of the capillaries in the CA1 was analyzed by multiplying the volume fraction of the capillaries in the CA1, V_V_ (cap/_CA1_), by the CA1 volume (V_(CA1)_).

### Total Surface Area of the Capillaries in CA1

Every captured photograph was randomly placed by grids of test lines (Figure 1D). The surface area density of the capillaries in the CA1 was calculated using the following formula (Shao et al., 2010):

(4) $${\text{S}}_{\text{V}}(\text{cap}{/}_{\text{CA1}})=2\times \sum \text{I}({\text{cap}}_{\left(\text{CA1}\right)})/\sum \text{L}$$

The total surface area of the capillaries in the CA1 was analyzed by multiplying SV (cap/_(CA1)_) by the CA1 volume (V_(CA1)_).

The total length, total volume and total surface area of the capillaries in the DG region were estimated using the same methods described above.

### Statistics

Data are expressed as the mean ± standard deviation (SD). Statistical analysis was performed for each of the dependent variables on the groups using analysis of variance (ANOVA). Group differences for these parameters were assessed using statistical product and service solutions software, with *p* < 0.05 as the criterion for statistical significance.

## Results

### Running Exercise Does Not Alter the Rats’ Body Weight in the CUS/Running Group

**Figure 2:**
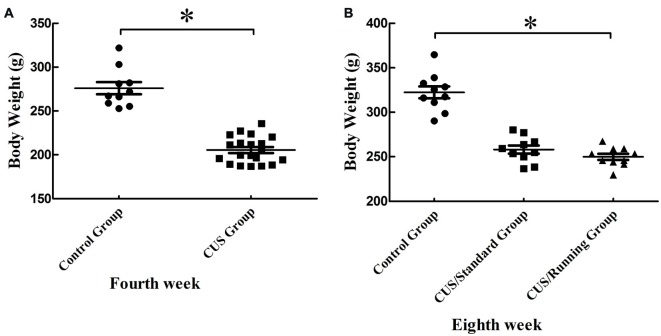
Changes of body weight before and after running exercise. **(A)** The comparison of body weight between the CUS group and control group in the procedure of CUS stimuli in the fourth week. **(B)** Effect of running exercise on the body weight among the three groups. *Indicates *p* < 0.05, compared with the control group.

After 4 weeks of CUS stimuli, the body weight significantly decreased in the CUS group compared with that in the control group (*p* < 0.05; Figure 2A). However, 4 weeks of running exercise did not increase the body weight in the CUS/Running group. The body in the CUS/Running group was still lower than that in the control group (*p* < 0.05; Figure 2B).

### Running Exercise Increases the Sucrose Consumption in the CUS/Running Group

**Figure 3:**
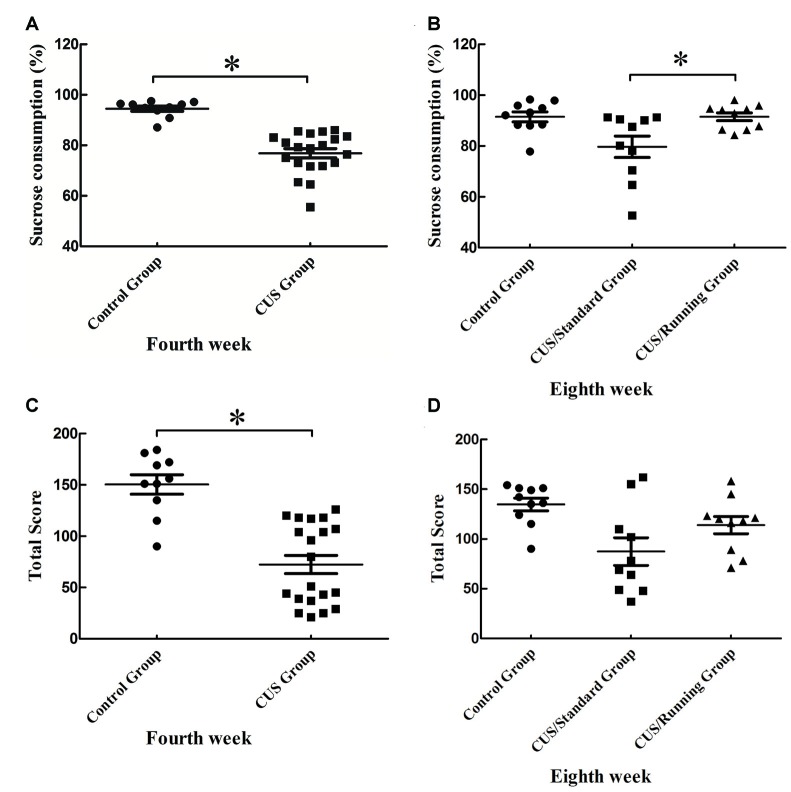
Changes of behavior tests before and after running exercise. **(A,C)** The results of sucrose consumption and open field test in the CUS group and control group after the procedure of CUS stimuli. **(B,D)** The results of sucrose consumption and open field test among the control group, CUS/Standard group and the CUS/Running group after running exercise. *Indicates *p* < 0.05.

After 4 weeks of CUS stimuli, the percentage of sucrose consumption in the CUS group ((76.9 ± 8.16) %) was significantly lower than that in the control group ((94.5 ± 3.24) %; *p* < 0.05; Figure 3A). This finding indicates that the construction of the depression model was successful. After the 4-week running period, the percentage of sucrose consumption in the CUS/Running group was elevated ((91.5 ± 4.83) %) and much higher than that in the CUS/Standard group ((79.7 ± 13.3) %) (*p* < 0.05; Figure 3B). The sucrose consumption test reflects the anhedonia. Therefore, our results demonstrated that 4 weeks of running exercise reversed depressive behaviors in rats. However, the results of another behavior test, the open field test, showed that CUS stimuli could significantly reduce the total score in the CUS group (*p* < 0.05; Figure 3C), but running exercise could not largely increase the total score in the CUS/Running group (Figure 3D).

### Running Exercise Significantly Increases the Volume of CA1 and DG Regions

**Figure 4:**
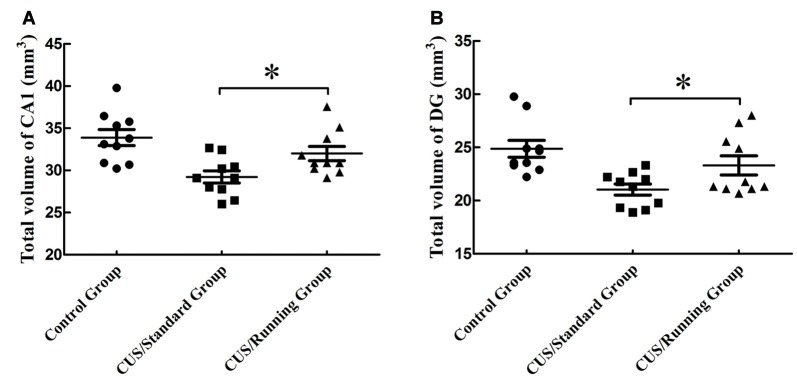
Influence of running exercise on the total volume in the CA1 and DG regions. **(A)** Comparison of the volume (mean ± SD) in CA1 among the control group, CUS/Standard group and the CUS/Running group. **(B)** Comparison of the volume (mean ± SD) in the DG region among the control group, CUS/Standard group and the CUS/Running group. *Indicates *p* < 0.05.

The accurate results of CA1 total volume were 33.9 ± 3.01 mm^3^ in the control group, 29.2 ± 2.27 mm^3^ in the CUS/Standard group and 32.0 ± 2.66 mm^3^ in the CUS/Running group. Statistical analysis showed that running exercise significantly increased the CA1 total volume in the CUS/Running group compared with that in the CUS/Standard group (Figure 4A).

Similarly, the accurate results of DG region total volume were 24.9 ± 2.52 mm^3^ in the control group, 21.0 ± 1.62 mm^3^ in the CUS/Standard group and 23.3 ± 2.84 mm^3^ in the CUS/Running group. The total volume of the DG region was significantly increased in the CUS/Running group compared with that in the CUS/Standard group (Figure 4B).

### Changes in the Total Length, Total Volume and Total Surface Area of the Capillaries in CA1

**Figure 5:**
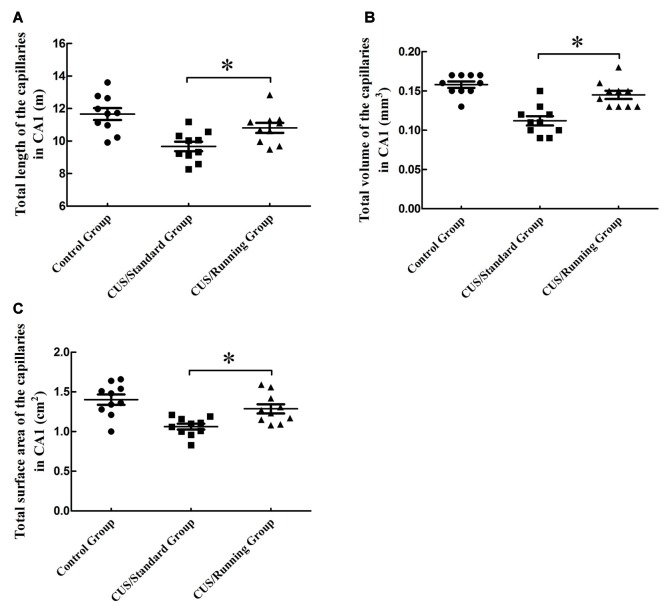
Influence of running exercise on the capillary parameters in the CA1. **(A)** Comparison of the capillaries’ total lengths in CA1 among the control group, CUS/Standard group and the CUS/Running group. **(B)** Comparison of the capillaries’ total volume in CA1 among the control group, CUS/Standard group and the CUS/Running group. **(C)** Comparison of the capillaries’ total surface area in CA1 among the control group, CUS/Standard group and the CUS/Running group. *Indicates *P* < 0.05.

The total lengths of the capillaries in the CA1 were 11.7 ± 1.15 m in the control group, 9.67 ± 0.92 m in the CUS/Standard group and 10.8 ± 0.976 m in the CUS/Running group. Running exercise significantly increased the total length of the capillaries in the CA1 in the CUS/Running group compared with that in the CUS/Standard group (Figure 5A).

The total volumes of the capillaries in the CA1 were 0.157 ± 0.0130 mm^3^ in the control group, 0.111 ± 0.0180 mm^3^ in the CUS/Standard group and 0.144 ± 0.0160 mm^3^ in the CUS/Running group. Running exercise significantly increased the total length of the capillaries in the CA1 in the CUS/Running group compared with that in the CUS/Standard group (Figure 5B).

The total surface areas of the capillaries in the CA1 were 1.40 ± 0.204 cm^2^ in the control group, 1.06 ± 0.117 cm^2^ in the CUS/Standard group and 1.29 ± 0.184 cm^2^ in the CUS/Running group. Running exercise significantly increased the total length of the capillaries in the CA1 in the CUS/Running group compared with that in the CUS/Standard group (Figure 5C).

However, both of the above three parameters in CA1 showed no differences between the control group and CUS/Running group (*p* > 0.05).

### Changes in the Total Length, Total Volume and Total Surface Area for the Capillaries in the DG Region

**Figure 6:**
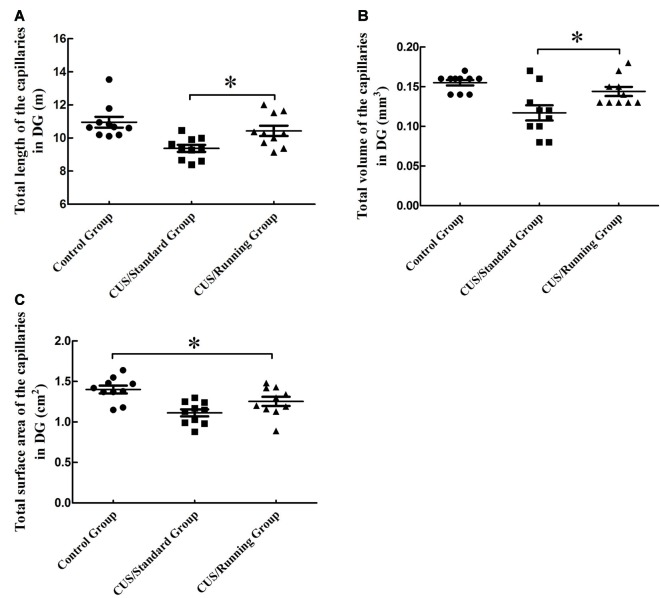
Influence of running exercise on the capillary parameters in DG region. **(A)** Comparison of the capillaries’ total lengths in DG region among the control group, CUS/Standard group and the CUS/Running group. **(B)** Comparison of the capillaries’ total volume in the DG region among the control group, CUS/Standard group and the CUS/Running group. **(C)** Comparison of the capillaries’ total surface area in the DG region among the control group, CUS/Standard group and the CUS/Running group. *Indicates *P* < 0.05.

The total lengths of the capillaries in the DG region were 10.9 ± 1.03 m in the control group, 9.38 ± 0.688 m in the CUS/Standard group and 10.4 ± 0.979 m in the CUS/Running group. Running exercise significantly increased the total length of the capillaries in the CA1 in the CUS/Running group compared with that in the CUS/Standard group (Figure 6A).

The total volumes of the capillaries in the DG region were 0.155 ± 0.0110 mm^3^ in the control group, 0.115 ± 0.0300 mm^3^ in the CUS/Standard group and 0.143 ± 0.0180 mm^3^ in the CUS/Running group. Running exercise significantly increased the total length of the capillaries in the DG region in the CUS/Running group compared with that in the CUS/Standard group (Figure 6B).

The total surface areas of the capillaries in the DG region were 1.40 ± 0.151 cm^2^ in the control group, 1.11 ± 0.135 cm^2^ in the CUS/Standard group and 1.25 ± 0.177 cm^2^ in the CUS/Running group. Statistical analysis showed no difference between the CUS/Running group and the CUS/Standard group, but the total surface area of the capillaries in the CUS/Running group was still lower than that in the control group (Figure 6C), which means running exercise had no significant effect on this parameter in the DG region.

## Discussion

Our study displayed that the preference of sucrose consumption in CUS/Running group was higher than that in the CUS/Standard group and reached normal levels when compared with the control group. Our results further confirmed that running had a therapeutic effect on the depression symptom. This research simulated the process of the development of human depression using the CUS depression model. Our study showed that the preference of sucrose consumption and the open-field score were decreased significantly in the CUS group after 4 weeks, which indicated that the depression model was established successfully. Running exercise, which is a safe, economic and simple means of behavior, has been successfully applied to the treatment of various diseases (Weuve et al., 2004; Ravaglia et al., 2008). Greist et al. (1979) reported that running exercise has the same therapeutic effect on depression compared with the traditional psychological treatment for the first time. Recently, clinical studies and animal experiments also demonstrated that running exercise is conducive to the rehabilitation of depression (Zheng et al., 2006; Ensari et al., 2014). However, Chalder et al. (2012) found that the addition of a facilitated physical activity intervention to usual care did not improve depression outcome or reduce use of antidepressants compared with usual care alone. We speculated that the following reasons might partly explain the different outcomes. First, Beck depression inventory scores of 361 adults aged 18–69 in the study of Chalder et al. (2012) are 14 or more. These Beck depression inventory scores indicated that the depression symptoms of the participants in their study were moderate or severe with different types (Beck et al., 1996). However, it is generally believed that running exercise has a positive effect on mild or moderate depression alone (McCurdy et al., 2017). Second, the intensity and duration of running exercise can be easily controlled in animal study. However, in human study, as Chalder et al. (2012) claimed in the discussion part, the basis of participants’ interest or motivation to engage in physical activity were not taken in to account. In human study, physical activity is notoriously difficult to measure and the self-reported assessment could have been biased by knowledge of the treatment allocation. To some degree, the participants’ knowledge of their treatment allocation influenced their responses. Therefore, we thought that the actual amount and time of exercise in the study of depression patients might be different from those in the regular exercise studies on the animals with the depression-like behaviors, which might have important impact on the overall results.

The hippocampal formation is composed of hippocampal gyrus (CA1, CA2 and CA3), DG and subiculum. The CA1 and DG are two significant parts of the hippocampal formation (Yaghmaei et al., 2009). With a deeper understanding of the pathogenesis of depression, researchers found that the whole brain volume in depression patients is not significantly different from that in normal subjects, but the hippocampus volume is significantly lower (Nifosi et al., 2010; Ahdidan et al., 2011). After 4 weeks of running, the volumes of the CA1 area and DG region are significantly increased in the CUS/Running group compared with those in the CUS/Standard group. However, the exact cellular mechanism behind the volume changes remains unknown. Degenerative changes in capillary seem to be one of the initial factors of nervous system diseases (Riddle et al., 2003). The incomplete function and structure of capillary can affect the support of oxygen and blood, leading to lower metabolism and chronic hypoperfusion (Farkas and Luiten, 2001).

With the development of imaging technology, researchers make dynamic observation of the blood flow in living brain, the chemical activity and metabolism of neuron systems, which provide much more support for the study of the vascular mechanism of depression (Niell and Smith, 2004). In the process of the treatment of depression, the researchers found that the vascular disease has also been improved (Otte et al., 2007) and augmentation of antidepressant therapy with vasodilators leads to greater depression reduction and lower rates of recurrence (Farkas and Luiten, 2001). These studies indicated that depression and cardiovascular diseases may have a common mechanism. Based on the above research results, we presumed that improving blood vessels may be a way of treating depression. Black et al. (1990) found that running exercise led to an increase in vascular density in the paramedian lobule of adult rats. After running exercise, the vascular density increased because of the metabolic need (Isaacs et al., 1992). However, most of these studies only observed blood flow changes in the brain or in a region of the brain after exercise using an imaging technique or other techniques. A capillary is a three-dimensional structure, and the distribution in the brain is extremely irregular; thus, different results could be obtained either from different biopsy parts or different directions. Moreover, the density value, which is the two-dimensional plane information, cannot reflect the real total blood capillary. Modern stereological methods are used to obtain accurate three-dimensional structure morphometric features (Tang et al., 2009), which provide good tools for the precise quantitative date of the capillaries. Using the stereological methods, we obtained accurate quantitative results of capillaries parameters in the CA1 and DG regions after running exercise, which provided a basic parameter for the treatment of depression.

In summary, our experiment accurately measured the basic parameters of hippocampus capillaries in CUS rats after running exercise, such as the total volume, total length and total surface area of capillary. Our result was consistent with the theory of “vascular depression”. It demonstrated that the depression rats had a change in the vascular structure and running exercise was an effective treatment for depression symptoms through reversing the decline of the parameters of the capillaries in the hippocampus. Therefore, drugs and behavior, which can influence capillaries, might be a new direction for the treatment of depression in the future.

## Author Contributions

All authors have full access to all research data and are responsible for the accuracy of data integrity and data analysis. LC, CT and YT: conception and design of the article. FW, YG and CH: performance of animal experiment and behavior tests. YZ, LJ and CZ: immumohistochemical staining. LC: analysis of the data. LC, CT and YT: writing and editing of the article.

## Conflict of Interest Statement

The authors declare that the research was conducted in the absence of any commercial or financial relationships that could be construed as a potential conflict of interest.
